# Farmers’ Houses

**Published:** 1866-03

**Authors:** 


For March, 1866.

### FARMERS' HOUSES.

Where to build and what shall be the plan of the house, are questions which have to be decided every year by thousands and thousands of enterprising farmers all over the country; either young men just married, who are about "opening" a farm in the boundless West, or by men more advanced in life, who, having done well, have decided to treat themselves and their faithful wives to a new and a better house than the one in which they have lived and striven so long and so well together.

In either case it is of the first consequence and is necessarily the first step to be taken, after having decided to build, to fix upon an answer to the question,

### WHERE SHALL I BUILD?

Upon the wise decision of this important inquiry depends, to a greater or less extent, the health, the consequent happiness, and eventual success in life, of every young farmer. It has been the experience of tens of thousands who began life hopefully, and who went to work with willing and brave hearts to "clear" a farm and make it a home for life for themselves and families, that they did well until sickness came, under which their strength and energy wilted away like a flower without water; they fell behindhand, lost their energy, ran in debt, and finally settled down in the poor ambition of only meeting their expenses from month to month; their idea of getting ahead having been abandoned forever.

It is demonstrably true, that the difference of a few hundred yards, of a dozen rods sometimes, in locating a dwelling for a family, is precisely the difference between its extinction, in a few years, by disease, and its prosperity, its health, and a large family of industrious manly sons, and of refined, educated, and notable daughters. A citizen of New-York purchased a beautiful building site for a country residence, and after spending two years and a large amount of money in preparing it for the reception of his wife, children, and servants, he moved into it. Every body was delighted with the "prospect" which it afforded, of river and field and woodlands and distant mountains. With autumn, came chills and fevers among his servants. He abandoned it, and never occupied it afterward, being wholly unwilling that his family should live where such a disease was possible.

A publishing house in this city erected a private residence in the country, at an expense of over thirty thousand dollars; it could be seen for many miles around; while its spacious piazzas afforded near and distant views, which delighted every visitor. During the very first year such a deadly pestilence broke out among the inmates, that it was at once abandoned, and was eventually "sold for a song." It is now known by residents on the banks of the Hudson as "Blank's Folly."

A wealthy and retired citizen of New-York built for himself a splendid mansion up-town, about four years ago, anticipating that it would be his home for life. He had occupied it but a short time, when one by one of the members of his family were taken sick. A strict examination discovered the fact that the house had been erected over a "filling," the emanations from which constantly ascending, impregnated every room in the building with deleterious gases; it was at once abandoned for another home.

The hospitals and barracks in and near Bengal are now almost useless, having been built in a locality utterly unfitted for human habitations, as far as health was concerned; their erection cost the British government sixty-five millions of dollars. This great waste of money might have been altogether avoided by the application of a very limited knowledge of the causes of disease.

From official papers presented to the British government, it is shown that of each hundred British soldiers in India, ninety-four disappear from the ranks before the age of thirty-five years, when, from military returns, it is known that "the average standard for health for Europeans in India, would compare with that existing anywhere else in the civilized world, if the known sources of disease were dried up." It is admitted, that in forty years, one hundred thousand men might have been saved, "*if proper localities had been chosen for their dwellings!*"

During the official investigations as to the causes of so much sickness and death at the National Hotel in Washington City some years ago, it was shown that there was no unusual sickness in any of the houses across the street, and that the causes of the disease were under the building itself. The symptoms in some persons were so malignant and virulent, it became the general conviction that they were the result of poison having been designedly introduced into the food. This proves the truth of the assertion already made, that the difference of a few feet in the locality of two buildings, is the difference sometimes between life and death. These things being so, it is a matter of personal happiness and pecuniary interest to every farmer who contemplates building a house, which is to be a home for himself and family, probably as long as he lives, to possess himself of such information as to enable him to ascertain certainly, why are certain localities so prejudicial to the health of families residing therein? or, in other words, what is the agent which causes disease in this mysterious manner? It may seem discouraging at first view to state that this destructive agency is as invisible as the viewless wind; at the same time it will afford encouragement to be assured that its nature is known, as also some of the laws by which it is regulated; and that by an easy attention to them, the Samson may be shorn of his locks; and the great destroyer may either be avoided, or rendered as harmless as the gentlest touch of infancy.

The name of this perfectly remorseless destroyer of human life is

"MIASM,"

From a Greek word which means *emanation;* that is, ARISING FROM; because it comes up from the surface of the earth. It is a short word, but it brings weary sickness and agonizing death to hundreds of thousands every year; it will bring sickness and death, sooner or later, to many a reader of this article; but a sickness and death which could have been avoided.

MIASM is the principal cause of nearly every "epidemic" disease; that is, of every sickness which "falls upon the people;" attacking numbers in any community, such as fever and ague, diarrhea, dysentery, cholera, bilious, intermittent, congestive and yellow fevers. But it is gratifying to know that it is an avoidable cause of disease. Money and wisely directed efforts can banish it from almost any locality. All that is needed is to know the laws of miasm, and wisely adapt ourselves to them.

In 1860, one of the daily papers of New-Orleans stated: "The

